# Molecular and cellular mechanisms of the effects of Propolis in inflammation, oxidative stress and glycemic control in chronic diseases

**DOI:** 10.1186/s12986-020-00485-5

**Published:** 2020-08-12

**Authors:** Naseh Pahlavani, Mahsa Malekahmadi, Safieh Firouzi, Daryoush Rostami, Alireza Sedaghat, Ahmad Bagheri Moghaddam, Gordon A. Ferns, Jamshid Gholizadeh Navashenaq, Reza Reazvani, Mohammad Safarian, Majid Ghayour-Mobarhan

**Affiliations:** 1 Student Research Committee, Mashhad University of Medical Sciences, Mashhad, Iran; 2 Department of Nutrition, Faculty of Medicine, Mashhad University of Medical Sciences, Vakil Abad Blvd., Opposite to Mellat Park, Mashhad, 99199-91766 Iran; 3 Department of Anesthesia, School of Paramedical Sciences, Zabol University of Medical Sciences, Zabol, Iran; 4 Cardiac Anesthesia Research Center, Mashhad University of Medical Sciences, Mashhad, Iran; 5 Department of Internal Medicine and Critical Care, Faculty of Medicine, Mashhad University of Medical Sciences, Mashhad, Iran; 6 Brighton & Sussex Medical School, Division of Medical Education, Falmer, Brighton, Sussex, BN1 9PH UK; 7 Noncommunicable Diseases Research Center, Bam University of Medical Sciences, Bam, Iran; 8 Metabolic Syndrome Research Center, Faculty of Medicine, Mashhad University of Medical Sciences, Vakil Abad Blvd., Opposite to Mellat Park, Mashhad, 99199-91766 Iran; 9 Cardiovascular Research Center, Mashhad University of Medical Sciences, Mashhad, Iran

**Keywords:** Propolis, Inflammation, Oxidative stress, Chronic disease, Glycemic control

## Abstract

Propolis is a sticky, resinous material gather from plants and is blended with wax and other constituents. It is reported to have anti-inflammatory, anti-oxidative and blood glucose-lowering properties. This review aims to summarise evidences for the cellular and molecular mechanism of Propolis in inflammation, oxidative stress, and glycemic control. Propolis stimulate the production and secretion of anti-inflammatory cytokines and to inhibit the production of inflammatory cytokines and due to its various antioxidant and poly-phenolic compounds may has a role in control and treating some of the chronic diseases. Most studies have shown that Propolis may affect metabolic factors including plasma insulin levels, and it has proposed that it could be used in the prevention and treatment of T2D Mellitus. In general, to demonstrate the definite effects of Propolis on chronic diseases, more studies are required using larger sample sizes and various doses of Propolis, using better characterized and standardized agents.

## Introduction

Propolis is a sticky, resinous material that bumble bees (*Apis mellifera* L.) gather from different plants and blend with wax and other constituents [1]. This material is collected by worker bees from the leaf buds of numerous tree species such as pine, alder, palm birch, poplar, and willow. The term Propolis derives from the Greek pro (for ‘before’, ‘at the passage to’) and polis (‘society’ or ‘city’) and means a substance produced by the hive [2]. Propolis has been used in complementary and alternative medicine in the past decades. There are over 300 potentially active ingredients in Propolis, which include comarins, phenolic aldehyde, steroids, amino acids and polyphenols [3]. Due to its potential medicinal properties, Propolis has been used for many different purposes such as immune enhancement, antibacterial effects, anti-inflammatory properties, antitumor, and anti-oxidant effects for several years [4, 5]. Because of the increased prevalence of chronic diseases, for example type 2 diabetes (T2D, therapeutic strategies for the prevention and treatment of these diseases may be useful in reducing the societal burden of these problems [6, 7]. The general recommendation for controlling chronic diseases include medicinal interventions and lifestyle modification (increased physical activity and diet modification) [8]. But these therapeutic approaches have some limitations, such as side effects of medications and severe dietary restriction that may be difficult to achieve compliance, therefore interest in complementary therapies has increased with the use of natural compounds with the minimum complications [9–12]. In previous studies in animal models, and clinical studies it has been shown that Propolis can improve glycemic control [13, 14]. However these effects have not yet been conclusively proven, and the mechanism of Propolis effects has not been fully clarified.

The purpose of this paper was to review the previous studies of Propolis and its possible mechanisms in reducing inflammation, oxidative stress and glycemic indices in various diseases.

## Propolis features and its components

Propolis is a natural viscose material made by honey bees and derived from parts of some plants [15]. Various studies have shown that the composition of Propolis is largely influenced by the honeybee species, geographic zone, food sources, and plants that the honey bee has used Nevertheless, it has been shown that Propolis from different parts of the world, including the Netherlands, China, Brazil and Peru, have similar antioxidant and free radical scavenging properties [16, 17]. The active ingredients of Propolis include: hydrocarbons, minerals, terpenoids, polyphenols, vitamins, amino acids, and several other active components are different depending on the geographical location and the species of honey bee [18]. The composition of Propolis comprises: 30% wax and 50% viscose resin, pollen, other organic materials and essential oils, account for 25% of its compositions [19]. Propolis bioactive active ingredient maybe as much as 70%, of which 58% is polyphenols and 20% flavonoids [20]. Various studies have shown that the major ingredients of Propolis derived from different regions of the world, such as Taiwan, New Zealand, Croatia, Africa, China and South Korea, are largely similar, these results are based on the characterization of various Propolis compounds using high-performance liquid chromatography (HPLC) gas chromatography (GC) and mass spectrometry (MS) [21, 22]. The main constituents of Iranian Propolis, include aromatic acids and their esters (mainly benzoic acid, vanilliacid, ferulicacid, p-coumaricacid, and caffeicacid), alkaloids (including 12-azabicyclo [9.2.2] pentadeca-1 (14),11(15)-dien-13-one and oreophilin), terpenes (mainly 3-tetramethyl, germanicol), flavonoids (mainly included pinostrobinchalcone, osthole, 2′,4′,6′-trihydroxy chalcone, and 3-methyl-but-2- enoicacid,2,2- dimethyl-8-oxo-3), and fatty acids and their related esters (mainly oleic acid, palmitic acid, stearic acid, margaric acid, and eicosanoic acid) [16]. The main compound of Propolis that has biological effects is caffeic acid phenethyl ester, that the amount of which was 12 mg / g Propolis in one study [23].

## Antioxidant property of Propolis

Previous studies using 2,2-diphenyl-1-picrylhydrazyl (DPPH), ferric-reducing antioxidant power (FRAP), 2,2′-azino-bis 3-ethylbenzothiazoline-6-sulphonic acid (ABTS^+^), and oxygen radical absorbance capacity (ORAC) methods have demonstrated the antioxidant properties of Propolis [24–26]. Propolis antioxidant activity is similar in mechanism to synthetic antioxidant butylated hydroxytoluene and vitamin C [27]. Propolis has 30–200 mg (GAE)/g phenolic of gallic acid equivalents of dry weight and 30–70 mg (QE)/g flavonoid of quercetin equivalents [28]. The activity of DPPH free radical-scavenging of Propolis is about 20–190 μg/mL [25]. Actually, different types of Propolis based on the origin of its botanic and collecting season are reported [29]. According to the Bankova classification, six major kind of Propolis were identified: poplar Propolis, Brazilian green Propolis, birch propolis, red Propolis, Canarian Propolis and pacific Propolis, [29]. Each component of Propolis has their own biological activity. Generally, the types of compounds are the same in most types of propolis, but the amount is different [26]. Strong antioxidant property of the Brazilian green Propolis refers to its content of 3,5-dicaffeoylquinic acid, 3,4,5-tricaffeoylquinic acid, artepillin C and 4,5-dicaffeoylquinic acid [28]. European (Italy and Russia) Propolis compared to Brazilian propolis has greater amount of polyphenolic and thus antioxidant activity [30]. Antioxidant content of Propolis affected by various factors, like plant origin, bee species, temperature, geographic location, season variation and storage conditions [27, 31]. Moreover, the biochemical composition and bioavailability of Propolis extracts affected by the solvents agent used for the extraction [32]. Use of solvent with high ethanol concentration results in extraction of Propolis with more antioxidant content [28]. It shows polar solvents obtains more antioxidant activity than the nonpoplar agents however, type of Propolis sample also affects this issue.

## Molecular mechanism of inflammation

Inflammation is a protective response of immune cells and vascular tissue injuries stimuli such as damaged cells and pathogens [33]. Inflammation is a protective mechanism that may eliminate the harmful stimuli and leading to beginning the process of healing [33]. It can be summarised by the following processes: phagocyte emigration, accumulation of monocytes, neutrophils, macrophages and loss of tissue function [34]. During the process of inflammation, macrophages activated the release of pro-inflammatory cytokines including of tumor necrosis factor-α (TNF-α), Interleukin 1(IL-1) and Interleukin 6 (IL-6). These macrophages stimulate the translocation of nuclear factor-kappaB (NF-kB). NF- kB has a major and significant role in the stimulation of cytokines and inflammatory mediators [34–36]. The NF-*κ*B is critical mediator in induction of genes involved in apoptosis and also it is key mediator in expression of pro-inflammatory and inflammatory cytokines genes including of TNF-α, IL-1, IL-2, IL-6 and IL-8 [37]*.* Also, NF-kB stimulates the production of nitric oxide synthase (NOS) enzyme and NOS generates nitric oxide (NO) that is an inflammatory mediator [38]. NO produced in inflammatory and endothelial cells and it can lead to tissue damage and ultimatly these processes may lead to pain and inflammation [39, 40]. Activator protein-1 (AP-1) is another transcriptional factor that has a critical role in cellular functions such as apoptosis and proliferation. Also, during infection, AP-1 may act in concert with NF-*κ*B and stimulates the inflammatory response [41]*.* Also*,* phagocytic cells, mast cells and endothelial cells my generate important inflammatory mediators by using plasma membrane lipids. Cytoplasmic membrane phospholipids and some of enzymes activated several extra and intracellular phospholipases such as lipoxygenase (LOX) and cyclooxygenase (COX) which play a main role in eicosanoid acid and arachidonic acid (AA) metabolism and ultimately producing major inflammatory factors such as leukotrienes and prostaglandins [42]. All of these mediators play a critical role in inflammatory process. LOX enzyme converts arachidonic acid to leukotriene A4 and produces leukotrienes B4. COX-1 and COX-2 coverts arachidonic acid to prostaglandin H2 and produces prostaglandins, thromboxanes and prostacylins [42, 43].

## Role of Propolis in inflammation

Propolis is reported to be a strong anti-inflammatory agent [44]. In recent years, in vitro and in vivo studies have been performed on the Propolis effects on inflammation, though the molecular mechanism for this property is not known [44, 45]. Caffeic acid phenethyl ester (CAPE) is a major constituent of Propolis, which is derived from the honeybee hives and it has anti-inflammatory effects. CAPE is a potent modulator of AA and it prevents the release of AA from the cell membrane and inhibits gene expression of LOX and COX enzymes that are involved in the AA metabolism pathways [46]. In in vitro and in vivo condition, the ethanol extract of propolis inhibited leukotriene and prostaglandin production. The effect of propolis on COX may be in result of its flavonoids, which have been demonstrated to suppressed prostaglandin endoperoxide synthase [46]. It also suppresses the activation of COX-1, COX-2 and gene responsible for COX-2 expression [47].

In Jurkat cells, CAPE has been shown to inhibit the activation of NF-kB by limiting the formation of nuclear factor of activated T cells (NFAT)-DNA and NF-kB DNA complexes and in result retarding NF-kB-dependent transcription [45, 48, 49]. In CAPE treated Jurkat cells, there was limited transcriptional activity of a Gal4-p65 hybrid protein and binding of NF-κB to DNA and also CAPE-mediated prevention of binding with DNA and activity of NFAT transcription was seen [48].

It has also been demonstrated that CAPE inhibits the production of inflammatory cytokines and increases the production of anti-inflammatory cytokines, including IL-10 and IL-4 [50]. Furthermore, stimulated T-cells, it inhibited the synthesis of IL-2 and also gene transcription of IL-2 [48, 51]. One study showed that CAPE at a dose of 0.1–25 μg/ml suppresses the production of TNF-a and interleukin (IL)-8; it eventually retards the expression of NF-kB, COX-2 and AP-1 [52]. Also, CAPE decreases the infiltration of monocytes and neutrophils that these are inflammatory cells [53]. Another study showed that CAPE interrupt in the interaction of the ligand (LPS) with the receptor complex (TLR4/MD2) and therefore it inhibited the activation of Toll-like receptor 4 (TLR4). TLR4 receptor is dysregulated in chronic inflammatory diseases. Therefore, CAPE may be effective in inflammatory diseases [54].

### Propolis and its effect on APAP-induced liver injury

One animal study examined the preventive effects of ethanol extract of Brazilian green Propolis (EEBGP) on hepatocellular necrosis in rats and anti-inflammatory effect of its including the expression of inflammatory genes. In this study, 291 mg/kg/day EEBGP was administrated, for 1 week. The result of this study showed that administration of EEBGP for 1 week in diet before *N*-acetyl-*p*-aminophenol administration of APAP reduced the percentage of hepatocellular necrosis. A possible mechanism for the anti-inflammatory effect of EEBGP is moderation of the inflammatory process. EEBGP administration decreased the mRNA inflammatory cytokines expression including *IL-10* and *IL-1β* and as a result it lead to decreasing in the hepatocellular necrosis percentage [55].

### Propolis and its effect on gastrointestinal disease

An in vitro study on the formation and development of Giardia duodenalis trophozoites showed that Propolis inhibited formation and development of the trophozoites and prevented the attachment of these parasitic organisms to the epithelial cells [56]. This study demonstrated transformation of the pear-shaped cell and decrease beating frequency of flagellar in the trophozoites [56]. Another experimental study indicated the antihistaminergic, antiinflammatory, anti-*H. pylori* activities and antiacid, of Propolis that could be used for the treatment of gastric ulceration [57]. Artepillin C and another phenolic mixtures available in Brazilian Propolis clears free radicals and decrease the oxidative stress relevant to inflammation [57]. Treatment with Propolis attenuated the levels of LPS (lipopolysaccharide) and down-regulated the TLR4 pathway and expressions of inflammatory markers in muscle of experimental mice, and improved serum triacylglycerols and glucose levels, also decreased inflammatory cytokines and endotoxemia by inhibiting dysbiosis in mice experimented with a high-fat diet [58].

### Propolis and its effect on ulcerative colitis

The two major forms of Inflammatory bowel disease (IBD) are Crohn’s disease and ulcerative colitis (UC). Crohn’s disease and UC are relatively rare disorders, but they result in frequent use of health care resources. Crohn’s disease and UC share some clinical characteristics, including diarrhea, fever, weight loss, abdominal pain and anemia [59]. Transcription factor NF-κβ overexpressed in patients with UC [60]. In inflammatory phase, NF-κβ is up regulated by IL, TNF-α, chemokines, interferon, and DNA damaging agents [61]. In UC patients, due to the over stimulation of NF-κβ, inflammatory mediators levels of pro inflammatory cytokines, including IL-1β, IL-6, TNF-α and interferons increase [62]. One study showed that administration of 5,10, 20 μM CAPE for 2 h suppresses translocation of NF-κβ, either by blocking of NF-κβ or by inhibition of Iκβ degradation [63]. Fitzpatrick et al. showed that CAPE 30 mg/kg/day for 1 week administered to rats, suppressed the NF휅B pathway, induce the macrophages apoptosis and decreasing the production of pro inflammatory mediators [64]. Khan et al. investigated the effects of CAPE in a mice with acute colitis by intraperitoneal injection of CAPE 30 mg/kg/day. The result of study showed that pro-inflammatory cytokines levels in colon, including IL1-β, IL6, INF-γ, TNF-α, and IL10 were considerably increased in mice with colitis, as compared to healthy mice [65]. On the other hands, Flavonoids are the major active components of Propolis and many study investigated the role of these flavonoids in UC patient. One study showed that flavonoids from Propolis had anti-inflammatory properties. These flavonoids suppressed the activation of NF-κβ and also it inhibited inflammatory genes expression such as TNF-α and IL-6 [66]. Another ingredient of Propolis are quercetin flavonoids. One study in DDS-induced colitis in rat showed that quercetin inhibited the NF-κB pathway. In this study, quercetin with dose of 1, 10, 50 μM for 1 h are used. The result of this study showed that quercetin suppressed the NF-κB and as a result stimulated the nitric oxide synthase expression. But, the molecular mechanisms of these pathway are yet unknown [67].

### Propolis and its effect on cancer

Propolis has selective toxic effects on tumor cells so that it inhibits tumor cells and has low or no toxicity effects on normal cells [68]. The anticancer property of ethanol extract of Chinese Propolis at concentration of 25, 50, 100, and 200 *μ*g/mL was shown to have selective toxic properties, being dose and time dependent; ethanol extracts of Chinese Propolis had minor toxicity effect on normal cells at 100 *μ*g/mL [68]. The involved mechanisms mainly contain regulation of p53 proteins and ANXA7, inhibition of NF-κB, mitochondrial membrane potential regulating and ROS [68]. Galangin, one of the major flavonoid in Propolis notably caused apoptosis and prevented melanoma cells in vitro study [69]. Also Propolis prevent of cell proliferation via stimulating endoplasmic reticulum stress, caspase activity, apoptosis, and decreasing the potential of mitochondrial membrane [70]. Propolis has cytotoxic activity with inhibition of MCF-7 and HeLa cells in cervical and breast cancer, this study indicated Propolis has an anticancer property [71].

### Propolis and its effect on diabetes

Previous investigations have shown that in diabetes mellitus the production of inflammatory cytokines including IL-1 [72], IL-6 and TNF-α increase [73]. Propolis has potent anti-inflammatory effects and it can inhibit the levels of these mediators [74, 75]. One study in type 2 diabetes mellitus (T2DM) patients demonstrated that Propolis at a dose of 1000 mg/ day administration for 3 months, significantly decreased hs-CRP and TNF-α levels but had no significantly difference seen for the serum IL-1β and IL-6 levels [76]. In Zhao et al. study Propolis supplementation with dose 100 and 900 mg/day for 18 weeks, in T2DM patients, significantly decrease serum levels of TNF-α, but it increase serum levels of IL-1β and IL-6. As a result propolis is effective in improving antioxidant function in diabetes mellitus [77].

**Fig. 1:**
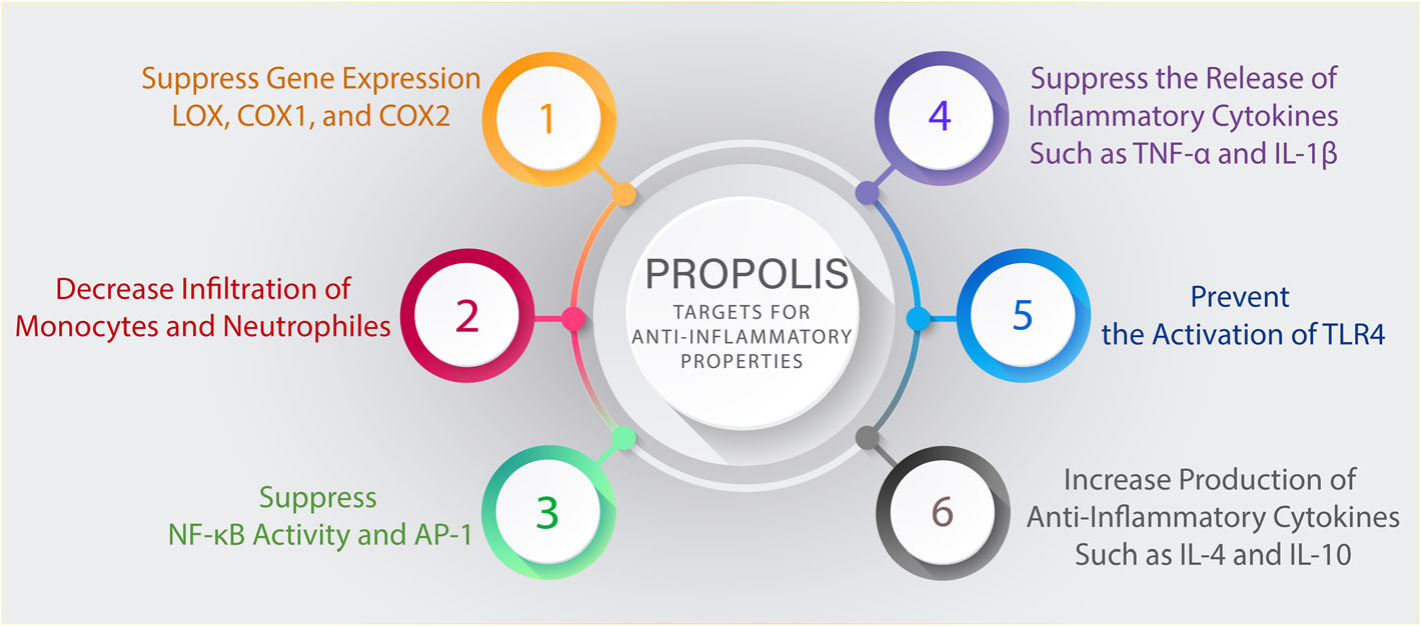
The probable anti-inflammatory effects of Propolis. Abbreviations: LOX; lipoxygenase. COX; Cyclooxygenase. TNF-α; Tumor Necrosis Factor Alpha. IL-1β; Interleukin 1β. IL-4; Interleukin 4. IL-10; Interleukin 10. AP-1;Activator protein 1.TLR4;Toll Like Receptor 4. NF-κB; nuclear factor kappa B

Propolis potential mechanisms for anti-inflammatory properties is shown in Fig. 1.

**Fig. 2:**
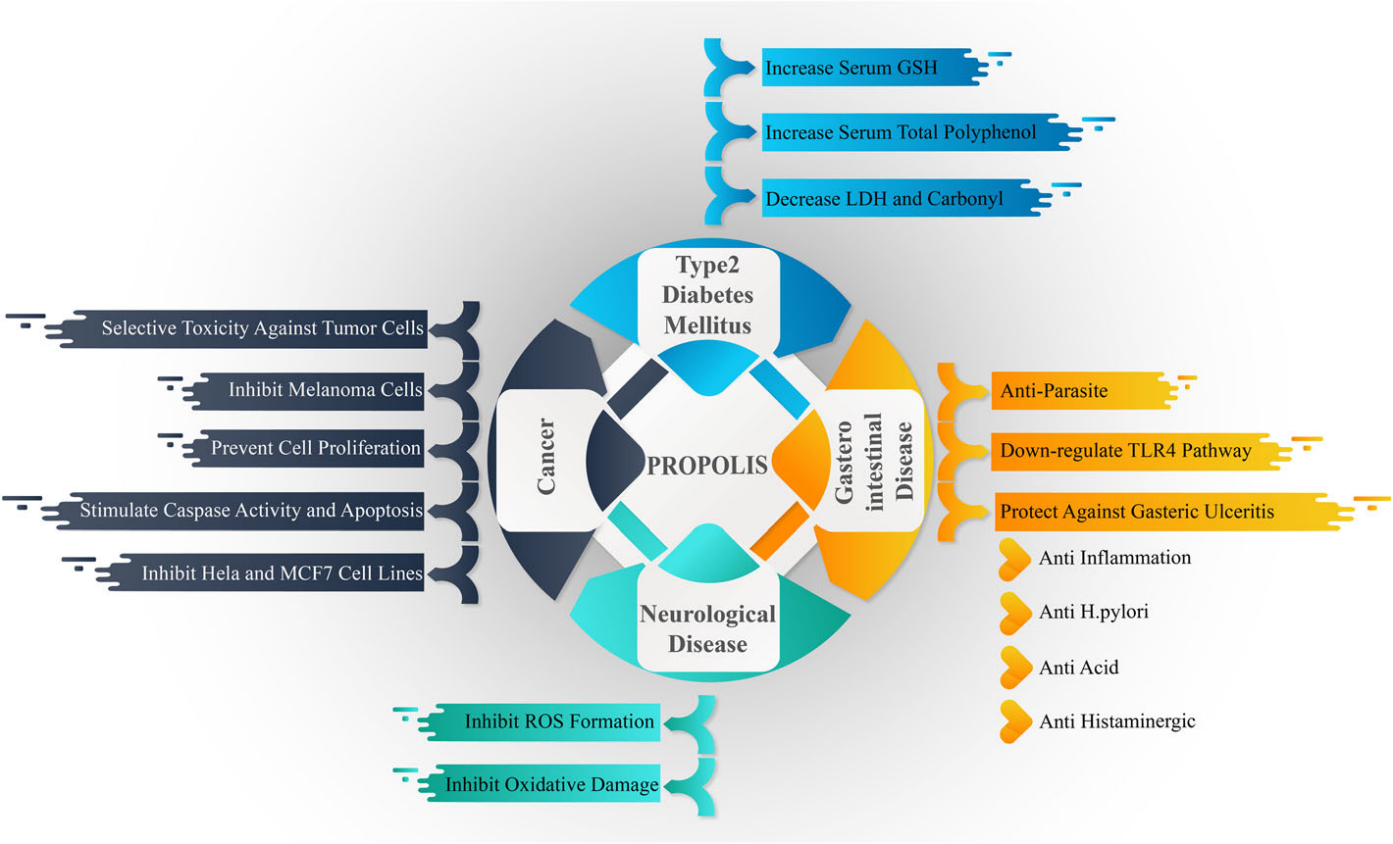
The probably antioxidant effects of Propolis in some chronic diseases. Abbreviations: GSH; Glutathione. LDH; Lactate dehydrogenase. MCF-7; human breast adenocarcinoma cell line. TLR4;Toll Like Receptor 4. ROS; Reactive Oxygen Species

In diabetic patients, the production of ROS and oxidative stress are elevated and antioxidant function is greatly reduced in some degree [78]. Also hyperglycemia by autoxidation of glucose, production of glycosylated products and polyol pathway, increases oxidative stress in this patients [79]. Moreover, dyslipidemia and chronic inflammation significantly enhance the oxidative stress in type 2 diabetes [78]. Propolis by reducing oxidative stress may protect vascular function against high glucose level [18, 77]. Animal study indicated that Brazilian green Propolis increased antioxidant balance in diabetic rats [80]. Clinical trials [18, 77] showed that Brazilian green Propolis remarkably increased serum GSH, and decreased serum carbonyls (oxidized proteins marker). In addition, serum total polyphenols were elevated. This indicated that the polyphenols of Propolis are bioavailable and after absorption acts as antioxidants [18, 77]. Antioxidant effects of Propolis in some diseases is shown in Fig. 2.

The potential effects of Propolis on oxidative stress and inflammation in clinical trial studies are shown at Table 1.

**Table 1:** Effects of propolis and it’s derivate on oxidative stress and inflammation in clinical trial studies

Author (year)	Country (Reference number)	Study design (Sex)	Participants numbers	Type and dose of Propolis administered	Duration (Mean age of subjects)	Outcome measures
Hesami S et al. (2019) [120]	Iran	Parallel RCT (M/F)	62 type 2 diabetic patients	Propolis capsule (500 mg) 3 times a day	8 weeks AGE (mean years (SD)): No information	S.R in Fructosamine level, the level of Ox-LDL S.I in catalase activity
Giammarinaro E et al. (2018) [121]	Italy	Parallel RCT (M/F)	40 Patients with gingivitis	Anti-oxidant gel formula contains propolis (dose not mentioned …)	3 months AGE (mean years (SD)): No information	S.R in Salivary oxidative stress
Javadi M et al. (2018) [122]	Iran	Parallel RCT (M)	60 men with asthenospermia	1500 mg (three capsules of 500 mg)	12 weeks AGE (mean years (SD)): No information	S.R in MDA. S.I in total antioxidant capacity
Gao W et al. (2018) [18]	China	Open-label CT study (M/F)	61 type 2 diabetes patients	1 capsule once daily each capsule: 900 mg	18-week (59.1 years)	S.I in GSH, Total polyphenols, total flavonoids and serum levels of IL-6 and S.R in LDH N.S in FRAP, SOD, GSHPx, MDA, carbonyls and serum levels of TNF-α and IL-1β
Zakerkish M et al. (2018) [81]	Iran	Parallel RCT (M/F)	94 patients with type 2 diabetes mellitus	2 capsules daily each capsule contain: 500 mg	90 days (55.14)	S.R in TNF-α and hs-CRP
Mujica V et al. (2017) [123]	Chile	Parallel RCT (M/F)	67 patients with metabolic disorder	15 drops of propolis 3% (Beepolis®)each time	90 days (46.4 years)	S.I in GSH levels, HDL S R in TBARS levels
Zhao L et al. (2016) [82]	China	Parallel RCT (M/F)	65 type 2 diabetes patients	1 capsule once daily each capsule: 900 mg	18-week (60.1 years)	S.I in GSH and total polyphenols, serum IL-1β and IL-6 S.R in serum carbonyls and LDH and serum TNF-α N.S in FRAP, SOD, GSH-Px, MDA or Ox-LDL
Afsharpour F et al. (2016) [35]	Iran	RCT(M/F)	62 patient with type 2 diabetes mellitus	3 capsules daily each capsule contain:500 mg	8 weeks(50.43)	S.R in CRP and TNF-α level
Fukuda T et al. (2015) [106]	Japan	RCT(M/F)	80 patient with type 2 diabetes mellitus	1 tablet once daily each tablet: 226.8 mg	8 weeks(63.31)	N.S in serum levels of TNF-α, hs-CRP and IL-6
Khayyal MT. et al. (2002) [124]	Egypt	RCT(M/F)	46 patients with mild to moderate asthma	1 capsule once daily each capsule: 200 mg	2 months	S.R in interleukin (IL)-6 and IL-8 and TNF-α S.I in The serum levels of IL-10

*Abbreviations*: *RCT* Randomized Clinical Trial, *M* Male, *F* Female, *S.R* Significantly Reduction, *S.I* Significantly Increased, *N.S* Not Significant difference, *TBARS* Thiobarbituric acid reactive substances, *GSH* Glutathione, *LDH* lactate dehydrogenase, *MDA* malondialdehyde, *FRAP* ferric-reducing antioxidant power, *GSH-Px* glutathione peroxidase, *Ox-LDL* oxidized low density lipoprotein, *SOD* superoxide dismutase, *TNF-α* Tumor Necrosis Factor-α, *Hs-CRP* High sensitive C-reactive Protein, *IL* interleukin

## Propolis and glycemic control

Type 2 diabetes (T2D) is a metabolic disorder specified by an increased blood sugar that results from inadequate insulin function and is associated with poor insulin release in insulin-sensitive tissues [83]. The increasing incidence of T2D in developed countries has increased the interest in research on natural compounds for prevention and control of this disease [14, 84]. The glycemic control goals established by the American Diabetes Association (ADA) are: fasting plasma glucose 80–130 mg/dL, glycosylated hemoglobin (A1C) < 7.0%, and casual plasma glycemia < 180 mg/dL [85]. In T2D, the antioxidant defense system is altered, and the inability of the body to scavenging free radicals may play a major role in tissue damage in diabetes [86]. Based on previous studies, it has been shown that Propolis supplementation can improve glycemic indices in subjects with T2D [8]. Previous studies have shown that Propolis has a positive effect on diabetes factors in animal studies [1, 13].

The control of hyperglycemia that increases the risk of pathogenicity and mortality due to complications of diabetes can be very helpful in improving of diabetic patient status [87]. Glycosylated hemoglobin (HbA1c) that correlated with long-term hyperglycemia, and fasting blood glucose (FBG) are the most important markers to predict the complications of diabetes and improve of these factors can be very effective in controlling T2D [88, 89]. HbA1c and FBG are effective predictors of micro-vascular complications in T2D patients [90]. It has been shown in some animal studies that Propolis can reduce FBG and HbA1c [81, 82, 91, 92]. Afsharpour et al. have shown that supplementation with Propolis (1500 mg/day during 8-week) could significantly decrease FBG and HbA1c in diabetic subjects [93]. Propolis, due to antioxidant properties, can also potentially help to reduce the complications and improve metabolic abnormalities (decrease of FBG and HbA1c levels) in T2D patients [29]. In a study done in Egypt on patients with T2D, Propolis supplementing with a dose of 400 mg/day for 6 months reduced FBG and HbA1c levels [14]. However, some studies have shown that Propolis has no significant effect on FBG and HbA1c [18, 94] for example in Zhao et al. study supplementation with 900 mg/day Propolis during 18-week had no significant reduction effect on FBG and HbA1c [77]. Inconsistencies in the Propolis effects on glycemic factors during the different studies probably due to: different doses of propolis supplementation, and different geographical origin of Propolis sample, and differences in the duration of intervention in patients [95].

Insulin resistance is one of the first features of type 2 diabetes, and so improving insulin resistance is one of the most important goals in the type 2 diabetes treatment [96]. In one animal study, encapsulated Propolis could improve insulin resistance in diabetic rats with increase insulin sensitivity mechanism [97]. In another study Propolis supplementation with dose 100 and 300 mg/kg, for 8 weeks in male Wistar rats improved insulin resistance by decreasing insulin plasma levels [98]. Many experimental studies showed that Propolis has beneficial effects on insulin sensitivity, blood glucose, HbA1c, and insulin levels in T2D animal models [92, 97, 99].

**Fig. 3:**
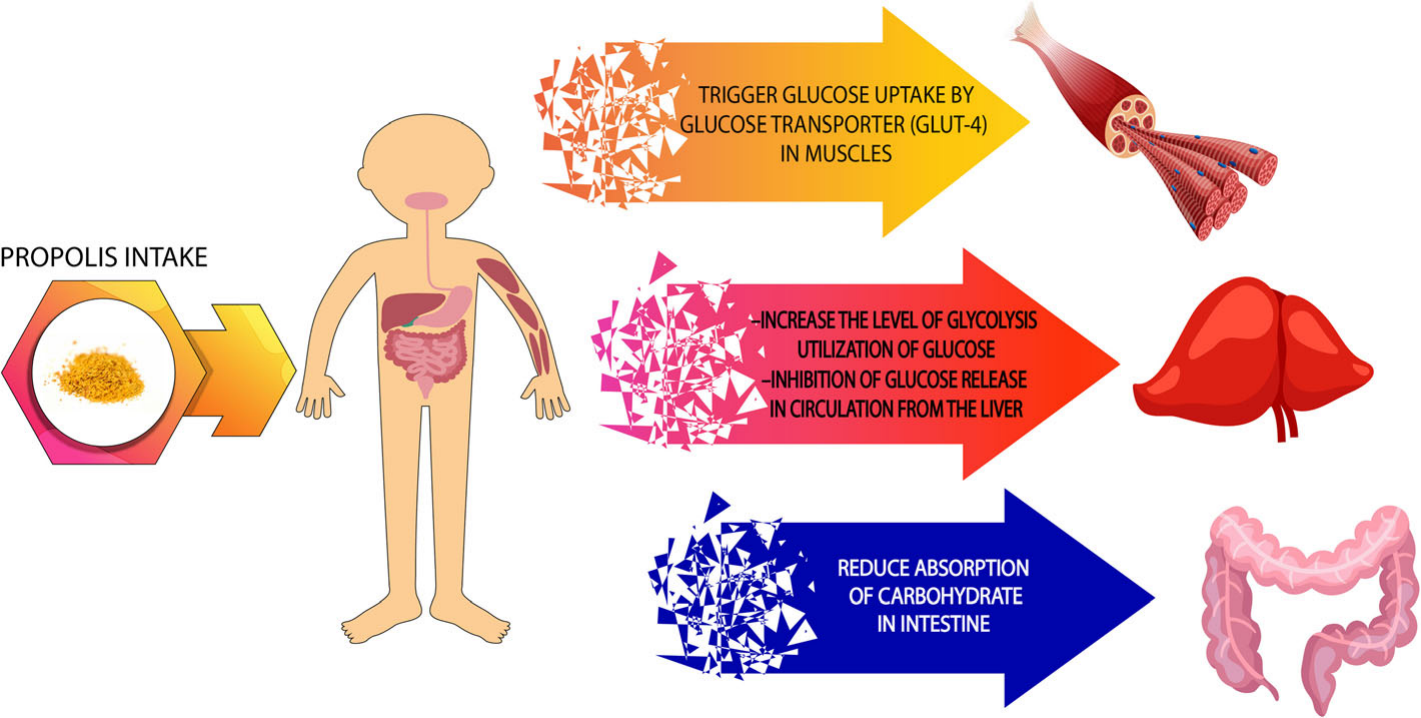
The probable mechanism of Propolis effects in glycemic indices control

In the Zakerkish et al. study administration of 1000 mg/day Iranian Propolis could increase insulin sensitivity during 3 months in T2D patients [76]. At the opposite point, supplementation with Brazilian green Propolis (226 mg/day during 8-week) did not effect on insulin levels and Homeostatic Model Assessment of Insulin Resistance (HOMA-IR) [94], these different effects are probably due to different doses of Propolis and the geographical location that Propolis collection. The Propolis effects on glysemic control in clinical trial studies are shown at Table 2. The production of reactive oxygen species induces hyperglycemia-activated electron-transport chain in mitochondria that is the essential mechanism linking between oxidative stress and pancreatic β cells dysfunctions [100]. Propolis mechanism for improving glycemic status is probably due to these reasons; 1- Increase glycolysis and glucose utilization in liver cells 2- Reduced carbohydrate intake in the gastrointestinal tract and intestinal cells 3- Activating of insulin-sensitive glucose transporters (GLUT-4) and glucose reabsorption by peripheral cells, such as skeletal muscle cells and 4- Inhibit glucose release from liver cells to blood circulation, these effects are likely to occur in doses ranging from 400 to 1500 mg of Propolis in the man [14, 76, 93, 101–103]. Furthermore, Propolis may have acted indirectly, by increasing β cell insulin secretion and improving insulin sensitivity [92]. The possible mechanisms of Propolis effects in glycemic indices control showed in Fig. 3. Studies have shown that Propolis, in addition to improving glycemic indices, can affect other metabolic factors including increased plasma insulin levels and other factors related with glycemic control [18, 77].

**Table 2:** Effects of propolis and it’s derivate on glycemic indices in clinical trial studies

Author (year)	Country (Reference number)	Study design (Sex)	Participants numbers	Type and dose of Propolis administered	Study Duration (Mean age of subjects)	Outcome measures
Zakerkish M et al. (2019) [81]	Iran	Parallel RCT (M/F)	94 type 2 diabetes patients	2 capsules daily each capsule contain: 500 mg	3-month (55.1 years)	S.R in HOMA-IR, Insulin, HbA1C, and 2 h-PPS levels in Propolis group compared with Placebo N.S in FPG between two groups
Afsharpour F et al. (2019) [35]	Iran	Parallel RCT (M/F)	62 type 2 diabetes patients	3 capsules daily each capsule contain: 500 mg	8-week (50.4 years)	S.R in FPG, HOMA-IR, Insulin, HbA1C, and 2 h-PPS levels in Propolis group compared with Placebo
Gao W et al. (2018) [18]	China	Open-label CT study (M/F)	61 type 2 diabetes patients	1 capsule once daily each capsule: 900 mg	18-week (59.1 years)	N.S in FPG, Insulin and HbA1C levels in Propolis group compared with Placebo
Samadi N et al. (2017) [105]	Iran	Parallel RCT (M/F)	66 type 2 diabetes patients	3 pills daily each pill contain: 300 mg	12-week (53.6 years)	S.R in FPG S.R in HbA1C level
El-Sharkawy H et al. (2016) [14]	Egypt	Parallel RCT (M/F)	50 type 2 diabetes patients	1 capsule once daily each capsule: 400 mg	6-month (50.1 years)	S.R in FPG S.R in HbA1C level
Zhao L et al. (2016) [82]	China	Parallel RCT (M/F)	65 type 2 diabetes patients	1 capsule once daily each capsule: 900 mg	18-week (60.1 years)	N.S in Serum glocuse, Insulin and HbA1C levels in Propolis group compared with Placebo
Fukuda T et al. (2015) [106]	Japan	Parallel RCT (M/F)	80 type 2 diabetes patients	1 Tablet daily each tablet contain: 226 mg	8-week (63.3 years)	N.S in HOMA-IR, Insulin and HbA1C levels in Propolis group compared with Placebo
Murata K et al. (2004) [117]	Japan	Case series study (M/F)	12 diabetic adult patients	Propolis Mixed with Mulberry Leaf Extract	30 days (44–74 years)	S.R in FPG S.R in HbA1C level

*Abbreviations*: *RCT* Randomized Clinical Trial, *M* Male, *F* Female, *G* Group, *S.R* Significantly Reduction, *N.S* Not Significant difference, *FPG* Fasting Plasma Glucose, *FBS* Fasting Blood Sugar, *2 h-PPS* 2 h Post Prandial Sugar, *HOMA-IR* Homeostatic Model Assessment of Insulin Resistance

## Conclusion

The present literature review suggested that Propolis may be beneficial in inflammatory conditions, oxidative stress and glycemic control in adults with chronic diseases. Propolis due to its various antioxidant and poly-phenolic compounds, as well as its lack of significant side effects and easy availability may has a role in control and treating some of the chronic diseases. In general, Inconsistency in the Propolis effects in different studies probably due to the heterogeneity of the Propolis components collected from different geographical locations and also given vary doses in different studies and smal sample size of studies, therefore more studies are needed to prove the definite effects of Propolis and to fully understand its molecular cellular mechanism.

## Data Availability

Not Applicable.

## References

[1] Fuliang H, Hepburn H, Xuan H, Chen M, Daya S, Radloff S (2005). Effects of propolis on blood glucose, blood lipid and free radicals in rats with diabetes mellitus. Pharmacol Res.

[2] Castaldo S, Capasso F (2002). Propolis, an old remedy used in modern medicine. Fitoterapia..

[3] de Castro PA, Savoldi M, Bonatto D, Barros MH, Goldman MHS, Berretta AA (2011). Molecular characterization of propolis-induced cell death in Saccharomyces cerevisiae. Eukaryot Cell.

[4] Lotfy M (2006). Biological activity of bee propolis in health and disease. Asian Pac J Cancer Prev.

[5] Pahlavani N, Sedaghat A, Bagheri Moghaddam A, Mazloumi Kiapey SS, Gholizadeh Navashenaq J, Jarahi L, et al. Effects of propolis and melatonin on oxidative stress, inflammation, and clinical status in patients with primary sepsis: Study protocol and review on previous studies. Clin Nutr ESPEN. 2019;33:125–31. 10.1016/j.clnesp.2019.06.007 31451248

[6] DeVol R, Bedroussian A, Charuworn A, Chatterjee A, Kim I, Kim S (2007). An unhealthy America: the economic burden of chronic disease. Santa Monica, CA: Milken Institute.

[7] Lam DW, LeRoith D (2012). The worldwide diabetes epidemic. Curr Opin Endocrinol Diabetes Obes.

[8] Karimian J, Hadi A, Pourmasoumi M, Najafgholizadeh A, Ghavami A. The efficacy of propolis on markers of glycemic control in adults with type 2 diabetes mellitus: a systematic review and meta-analysis. Phytother Res 2019 Jun;33(6):1616–1626. PubMed PMID: 30950136. Epub 2019/04/06. eng. 10.1002/ptr.6356 30950136

[9] Grunberger G (2017). Should side effects influence the selection of antidiabetic therapies in type 2 diabetes?. Curr Diabetes Rep.

[10] Yang J, Huang C, Wu S, Xu Y, Cai T, Chai S (2017). The effects of dipeptidyl peptidase-4 inhibitors on bone fracture among patients with type 2 diabetes mellitus: a network meta-analysis of randomized controlled trials. PLoS One.

[11] Kumar H, Kim I-S, More SV, Kim B-W, Choi D-K (2014). Natural product-derived pharmacological modulators of Nrf2/ARE pathway for chronic diseases. Nat Prod Rep.

[12] Rasad H, Entezari MH, Ghadiri E, Mahaki B, Pahlavani N (2018). The effect of honey consumption compared with sucrose on lipid profile in young healthy subjects (randomized clinical trial). Clin Nutr ESPEN.

[13] Zhu W, Chen M, Shou Q, Li Y, Hu F. Biological activities of Chinese propolis and Brazilian propolis on streptozotocin-induced type 1 diabetes mellitus in rats. Evid Based Complement Alternat Med. 2011;2011:1–8. 10.1093/ecam/neq025 PMC3135653 21785625

[14] El-Sharkawy HM, Anees MM, Van Dyke TE. Propolis improves periodontal status and glycemic control in patients with type 2 diabetes mellitus and chronic periodontitis: a randomized clinical trial. J Periodontol 2016 Dec;87(12):1418–1426. PubMed PMID: 27468795. Epub 2016/07/30. eng. 10.1902/jop.2016.150694 27468795

[15] Gabrys J, Konecki J, Krol W, Scheller S, Shani J. Free amino acids in bee hive product (propolis) as identified and quantified by gas-liquid chromatography. Pharmacol Res Commun 1986 Jun;18(6):513–518. PubMed PMID: 3749241. Epub 1986/06/01. eng. 10.1016/0031-6989(86)90146-3 3749241

[16] Ahangari Z, Naseri M, Vatandoost F. Propolis: chemical composition and its applications in Endodontics. Iran Endod J 2018 Summer;13(3):285–292. PubMed PMID: 30083195. Pubmed Central PMCID: PMC6064031. Epub 2018/08/08. eng. 10.22037/iej.v13i3.20994 PMC6064031 30083195

[17] Banskota AH, Tezuka Y, Adnyana IK, Midorikawa K, Matsushige K, Message D (2000). Cytotoxic, hepatoprotective and free radical scavenging effects of propolis from Brazil, Peru, the Netherlands and China. J Ethnopharmacol.

[18] Gao W, Pu L, Wei J, Yao Z, Wang Y, Shi T (2018). Serum antioxidant parameters are significantly increased in patients with type 2 diabetes mellitus after consumption of Chinese propolis: a randomized controlled trial based on fasting serum glucose level. Diabetes Ther.

[19] Kurek-Górecka A, Rzepecka-Stojko A, Górecki M, Stojko J, Sosada M, Świerczek-Zięba G (2014). Structure and antioxidant activity of polyphenols derived from propolis. Molecules..

[20] Sforcin JM (2016). Biological properties and therapeutic applications of propolis. Phytother Res.

[21] Li F, Awale S, Tezuka Y, Esumi H, Kadota S (2010). Study on the constituents of Mexican propolis and their cytotoxic activity against PANC-1 human pancreatic cancer cells. J Nat Prod.

[22] El-Guendouz S, Lyoussi B, Miguel MGC. Insight on propolis from Mediterranean countries chemical composition, biological activities, and application fields. Chem Biodivers. 2019;16(7):e1900094. 10.1002/cbdv.201900094 31099458

[23] Demestre M, Messerli S, Celli N, Shahhossini M, Kluwe L, Mautner V (2009). CAPE (caffeic acid phenethyl ester)-based propolis extract (Bio 30) suppresses the growth of human neurofibromatosis (NF) tumor xenografts in mice. Phytother Res.

[24] Betances-Salcedo E, Revilla I, Vivar-Quintana A, González-Martín M (2017). Flavonoid and antioxidant capacity of propolis prediction using near infrared spectroscopy. Sensors..

[25] Zhang C, Shen X, Chen J, Jiang X, Hu F (2017). Identification of free radical scavengers from Brazilian green propolis using off-line HPLC-DPPH assay and LC-MS. J Food Sci.

[26] Andrade JKS, Denadai M, de Oliveira CS, Nunes ML, Narain N (2017). Evaluation of bioactive compounds potential and antioxidant activity of brown, green and red propolis from Brazilian northeast region. Food Res Int.

[27] Bonamigo T, Campos JF, Alfredo TM, Balestieri JBP, Cardoso CAL, Paredes-Gamero EJ, et al. Antioxidant, cytotoxic, and toxic activities of propolis from two native bees in Brazil: Scaptotrigona depilis and Melipona quadrifasciata anthidioides. Oxidative Med Cell Longev. 2017;2017:1–12. 10.1155/2017/1038153 PMC5362732 28377794

[28] Kocot J, Kiełczykowska M, Luchowska-Kocot D, Kurzepa J, Musik I. Antioxidant potential of propolis, bee pollen, and royal jelly: possible medical application. Oxidative Med Cell Longev. 2018;2018:1–29. 10.1155/2018/7074209 PMC5954854 29854089

[29] Samadi N, Mozaffari-Khosravi H, Rahmanian M, Askarishahi M (2017). Effects of bee propolis supplementation on glycemic control, lipid profile and insulin resistance indices in patients with type 2 diabetes: a randomized, double-blind clinical trial. J Integr Med.

[30] Fabris S, Bertelle M, Astafyeva O, Gregoris E, Zangrando R, Gambaro A (2013). Antioxidant properties and chemical composition relationship of Europeans and Brazilians propolis. Pharmacol Pharm.

[31] Calegari MA, Prasniewski A, SILVA CD, Sado RY, Maia F, Tonial L (2017). Propolis from southwest of Parana produced by selected bees: influence of seasonality and food supplementation on antioxidant activity and phenolic profile. An Acad Bras Cienc.

[32] Narimane S, Demircan E, Salah A, Salah R. Correlation between antioxidant activity and phenolic acids profile and content of Algerian propolis: influence of solvent. Pak J Pharm Sci. 2017;30:1417–23. 29043991

[33] Liu T, Zhang L, Joo D, Sun S-C (2017). NF-κB signaling in inflammation. Signal Transduct Target Ther.

[34] Sigal LH, Ron Y (1994). Immunology and inflammation. Basic mechanisms and clinical consequences.

[35] Surh Y-J, Chun K-S, Cha H-H, Han SS, Keum Y-S, Park K-K (2001). Molecular mechanisms underlying chemopreventive activities of anti-inflammatory phytochemicals: down-regulation of COX-2 and iNOS through suppression of NF-κB activation. Mutat Res/Fundam Mol Mechanisms Mutagenesis.

[36] Baeuerle PA (1991). The inducible transcription activator NF-κB: regulation by distinct protein subunits. Biochimica et Biophysica Acta (BBA)-reviews on. Cancer..

[37] Barnes PJ, Karin M (1997). Nuclear factor-kappaB: a pivotal transcription factor in chronic inflammatory diseases. N Engl J Med.

[38] Xie Q, Kashiwabara Y, Nathan C (1994). Role of transcription factor NF-kappa B/Rel in induction of nitric oxide synthase. J Biol Chem.

[39] Baig MS, Zaichick SV, Mao M, de Abreu AL, Bakhshi FR, Hart PC (2015). NOS1-derived nitric oxide promotes NF-κB transcriptional activity through inhibition of suppressor of cytokine signaling-1. J Exp Med.

[40] Guzik T, Korbut R, Adamek-Guzik T (2003). Nitric oxide and superoxide in inflammation. J Physiol Pharmacol.

[41] Kopp EB, Ghosh S (1995). NF-kappa B and rel proteins in innate immunity. Adv Immunol.

[42] Salmon JA, Higgs GA (1987). Prostaglandins and leukotrienes as inflammatory mediators. Br Med Bull.

[43] Funk CD (2001). Prostaglandins and Leukotrienes: advances in eicosanoid biology. Science..

[44] Ying-Hua L, Wei Z, Fu-Liang H. Progress on anti-inflammatory effects and mechanism of Propolis. Natural Product Res Dev. 2012;24(6):856–9.

[45] Natarajan K, Singh S, Burke TR, Grunberger D, Aggarwal BB (1996). Caffeic acid phenethyl ester is a potent and specific inhibitor of activation of nuclear transcription factor NF-kappa B. Proc Natl Acad Sci.

[46] Mirzoeva OK, Calder PC. The effect of propolis and its components on eicosanoid production during the inflammatory response. Prostaglandins Leukot Essent Fat Acids 1996 Dec;55(6):441–449. PubMed PMID: 9014224. Epub 1996/12/01. eng. 10.1016/s0952-3278(96)90129-5 9014224

[47] Orban Z, Mitsiades N, Burke TR, Tsokos M, Chrousos GP (2000). Caffeic acid phenethyl ester induces leukocyte apoptosis, modulates nuclear factor-kappa B and suppresses acute inflammation. Neuroimmunomodulation..

[48] Zhao WX, Wang L, Yang JL, Li LZ, Xu WM, Li T (2014). Caffeic acid phenethyl ester attenuates pro-inflammatory and fibrogenic phenotypes of LPS-stimulated hepatic stellate cells through the inhibition of NF-kappaB signaling. Int J Mol Med.

[49] Chuu CP, Lin HP, Ciaccio MF, Kokontis JM, Hause RJ, Hiipakka RA (2012). Caffeic acid phenethyl ester suppresses the proliferation of human prostate cancer cells through inhibition of p70S6K and Akt signaling networks. Cancer Prev Res (Philadelphia, Pa).

[50] Moura SAL, Ferreira MAND, Andrade SP, Reis MLC, Noviello ML, Cara DC. Brazilian green propolis inhibits inflammatory angiogenesis in a murine sponge model. Evid Based Complement Alternat Med. 2011;2011:1–7. 10.1093/ecam/nep197 PMC3094767 20007259

[51] Lee JY, Choi HJ, Chung TW, Kim CH, Jeong HS, Ha KT (2013). Caffeic acid phenethyl ester inhibits alpha-melanocyte stimulating hormone-induced melanin synthesis through suppressing transactivation activity of microphthalmia-associated transcription factor. J Nat Prod.

[52] Abdel-Latif MM, Windle HJ, Homasany BS, Sabra K, Kelleher D (2005). Caffeic acid phenethyl ester modulates helicobacter pylori-induced nuclear factor-kappa B and activator protein-1 expression in gastric epithelial cells. Br J Pharmacol.

[53] Rajoo M, Parolia A, Pau A, Amalraj FD. The role of propolis in inflammation and orofacial pain: a review. Ann Res Rev Biol. 2014;4(4):651–64.

[54] Kim SY, Koo JE, Seo YJ, Tyagi N, Jeong E, Choi J (2013). Suppression of toll-like receptor 4 activation by caffeic acid phenethyl ester is mediated by interference of LPS binding to MD2. Br J Pharmacol.

[55] Tsuchiya Y, Sakai H, Hirata A, Yanai T. Brazilian green propolis suppresses acetaminophen-induced hepatocellular necrosis by modulating inflammation-related factors in rats. J Toxicol Pathol 2018 Oct;31(4):275–282. PubMed PMID: 30393431. Pubmed Central PMCID: PMC6206282. Epub 2018/11/06. eng. 10.1293/tox.2018-0027 PMC6206282 30393431

[56] Freitas S, Shinohara L, Sforcin J, Guimarães S (2006). In vitro effects of propolis on Giardia duodenalis trophozoites. Phytomedicine..

[57] Paulino N, Coutinho LA, Coutinho JR, Vilela GC, da Silva Leandro VP, Paulino AS (2015). Antiulcerogenic effect of Brazilian propolis formulation in mice. Pharmacol Pharm..

[58] Roquetto AR, Monteiro NES, Moura CS, Toreti VC, de Pace F, Santos AD (2015). Green propolis modulates gut microbiota, reduces endotoxemia and expression of TLR4 pathway in mice fed a high-fat diet. Food Res Int.

[59] Neurath MF. Cytokines in inflammatory bowel disease. Nat Rev Immunol 2014 May;14(5):329–342. PubMed PMID: 24751956. Epub 2014/04/23. eng. 10.1038/nri3661 24751956

[60] Atreya I, Atreya R, Neurath MF. NF-kappaB in inflammatory bowel disease. J Intern Med 2008 Jun;263(6):591–596. PubMed PMID: 18479258. Epub 2008/05/16. eng. 10.1111/j.1365-2796.2008.01953.x 18479258

[61] Lawrence T (2009). The nuclear factor NF-kappaB pathway in inflammation. Cold Spring Harb Perspect Biol.

[62] Schreiber S, Nikolaus S, Hampe J (1998). Activation of nuclear factor kappa B inflammatory bowel disease. Gut..

[63] Wang LC, Chu KH, Liang YC, Lin YL, Chiang BL (2010). Caffeic acid phenethyl ester inhibits nuclear factor-kappaB and protein kinase B signalling pathways and induces caspase-3 expression in primary human CD4+ T cells. Clin Exp Immunol.

[64] Fitzpatrick LR, Wang J, Le T (2001). Caffeic acid Phenethyl Ester, an inhibitor of nuclear factor-κB, attenuates bacterial peptidoglycan polysaccharide-induced colitis in rats. J Pharmacol Exp Ther.

[65] Khan MN, Lane ME, McCarron PA, Tambuwala MM (2018). Caffeic acid phenethyl ester is protective in experimental ulcerative colitis via reduction in levels of pro-inflammatory mediators and enhancement of epithelial barrier function. Inflammopharmacology..

[66] Funakoshi-Tago M, Okamoto K, Izumi R, Tago K, Yanagisawa K, Narukawa Y (2015). Anti-inflammatory activity of flavonoids in Nepalese propolis is attributed to inhibition of the IL-33 signaling pathway. Int Immunopharmacol.

[67] Comalada M, Camuesco D, Sierra S, Ballester I, Xaus J, Gálvez J (2005). In vivo quercitrin anti-inflammatory effect involves release of quercetin, which inhibits inflammation through down-regulation of the NF-κB pathway. Eur J Immunol.

[68] Xuan H, Li Z, Yan H, Sang Q, Wang K, He Q, et al. Antitumor activity of Chinese propolis in human breast cancer MCF-7 and MDA-MB-231 cells. Evid Based Complement Alternat Med. 2014;2014:1–11. 10.1155/2014/280120 PMC4055122 24963320

[69] Benguedouar L, Lahouel M, Gangloff S, Durlach A, Grange F, Bernard P, et al. Algerian ethanolic extract of propolis and galangin decreased melanoma tumour progression in C57BL6 mice. Ann Dermatol Vénér. 2015;142(6–7):S294. 10.2174/1871520616666160211124459 26863880

[70] Demir S, Aliyazicioglu Y, Turan I, Misir S, Mentese A, Yaman SO (2016). Antiproliferative and proapoptotic activity of Turkish propolis on human lung cancer cell line. Nutr Cancer.

[71] Diva AN, Pratami DK, Wijanarko A, Hermansyah H, Sahlan M. Effect of ethanolic propolis extract from *Tetragonula biroi* bees on the growth of human cancer cell lines HeLa and MCF-7. AIP Conference Proceedings. Florida: AIP Publishing; 2019.

[72] Maedler K, Fontana A, Ris F, Sergeev P, Toso C, Oberholzer J (2002). FLIP switches Fas-mediated glucose signaling in human pancreatic beta cells from apoptosis to cell replication. Proc Natl Acad Sci U S A.

[73] Hotamisligil GS, Arner P, Caro JF, Atkinson RL, Spiegelman BM. Increased adipose tissue expression of tumor necrosis factor-alpha in human obesity and insulin resistance. J Clin Invest 1995 May;95(5):2409–2415. PubMed PMID: 7738205. Pubmed Central PMCID: PMC295872. Epub 1995/05/01. eng. 10.1172/JCI117936 PMC295872 7738205

[74] Silva-Carvalho R, Baltazar F, Almeida-Aguiar C (2015). Propolis: a complex natural product with a plethora of biological activities that can be explored for drug development. Evid-based Complement Altern Med.

[75] Al Ghamdi AA, Badr G, Hozzein WN, Allam A, Al-Waili NS, Al-Wadaan MA, et al. Oral supplementation of diabetic mice with propolis restores the proliferation capacity and chemotaxis of B and T lymphocytes towards CCL21 and CXCL12 by modulating the lipid profile, the pro-inflammatory cytokine levels and oxidative stress. BMC Immunol 2015 Sep 15;16:54. PubMed PMID: 26370805. Pubmed Central PMCID: PMC4570673. Epub 2015/09/16. eng. 10.1186/s12865-015-0117-9 PMC4570673 26370805

[76] Zakerkish M, Jenabi M, Zaeemzadeh N, Hemmati AA, Neisi N (2019). The effect of Iranian Propolis on glucose metabolism, lipid profile, insulin resistance, renal function and inflammatory biomarkers in patients with type 2 diabetes mellitus: a randomized double-blind clinical trial. Sci Rep.

[77] Zhao L, Pu L, Wei J, Li J, Wu J, Xin Z (2016). Brazilian green propolis improves antioxidant function in patients with type 2 diabetes mellitus. Int J Environ Res Public Health.

[78] Aouacheri O, Saka S, Krim M, Messaadia A, Maidi I (2015). The investigation of the oxidative stress-related parameters in type 2 diabetes mellitus. Can J Diabetes.

[79] Maritim AC, Sanders RA, Watkins JB (2003). Diabetes, oxidative stress, and antioxidants: a review. J Biochem Mol Toxicol.

[80] Zhang N, Wu J, Gao W, Wei J, Pu L, Jiao C (2014). The comparative study of oxidative stress in rats with diabetes mellitus by propolis from different origins. Chin J Food Hyg.

[81] Oršolić N, Sirovina D, Končić MZ, Lacković G, Gregorović G (2012). Effect of Croatian propolis on diabetic nephropathy and liver toxicity in mice. BMC Complement Altern Med.

[82] Zhu W, Li YH, Chen ML, Hu FL (2011). Protective effects of Chinese and Brazilian propolis treatment against hepatorenal lesion in diabetic rats. Hum Exp Toxicol.

[83] King H, Aubert RE, Herman WH (1998). Global burden of diabetes, 1995–2025: prevalence, numerical estimates, and projections. Diabetes Care.

[84] Al-Hariri MT (2011). Propolis and its direct and indirect hypoglycemic effect. J Fam Community Med.

[85] Inzucchi SE, Bergenstal R, Buse J, Diamant M, Ferrannini E, Nauck M (2012). Management of hyperglycaemia in type 2 diabetes: a patient-centered approach. Position statement of the American Diabetes Association (ADA) and the European Association for the study of diabetes (EASD). Diabetologia..

[86] Lane TA, Lamkin GE, Wancewicz EV (1990). Protein kinase C inhibitors block the enhanced expression of intercellular adhesion molecule-1 on endothelial cells activated by interleukin-1, lipopolysaccharide and tumor necrosis factor. Biochem Biophys Res Commun.

[87] Viana MV, Moraes RB, Fabbrin AR, Santos MF, Gerchman F (2014). Assessment and treatment of hyperglycemia in critically ill patients. Rev Bras Ter Intensiva.

[88] Ghazanfari Z, Haghdoost AA, Alizadeh SM, Atapour J, Zolala F (2010). A comparison of HbA1c and fasting blood sugar tests in general population. Int J Prev Med.

[89] Azizi Soleiman F, Pahlavani N, Rasad H, Sadeghi O, Gohari MR (2013). The relationship between inflammation, oxidative stress, and metabolic risk factors in type 2 diabetic patients. Iran J Diabetes Obes.

[90] Juarez DT, Demaris KM, Goo R, Mnatzaganian CL, Smith HW (2014). Significance of HbA1c and its measurement in the diagnosis of diabetes mellitus: US experience. Diabetes, Metab Syndr Obes: Targets Ther.

[91] Abdulbasit A, Oladayo M, Olamide F, Olasile O, Babatunde I, Gbolahan B (2013). Effect of Nigerian propolis on glycemia, lipid profile, and oxidative stress markers in alloxan-induced diabetic rats. Pharmacologyonline..

[92] Oladayo MI (2016). Nigerian propolis improves blood glucose, glycated hemoglobin A1c, very low-density lipoprotein, and high-density lipoprotein levels in rat models of diabetes. J Intercultural Ethnopharmacol.

[93] Afsharpour F, Javadi M, Hashemipour S, Koushan Y, Haghighian HK (2019). Propolis supplementation improves glycemic and antioxidant status in patients with type 2 diabetes: a randomized, double-blind, placebo-controlled study. Complement Ther Med.

[94] Fukuda T, Fukui M, Tanaka M, Senmaru T, Iwase H, Yamazaki M (2015). Effect of Brazilian green propolis in patients with type 2 diabetes: a double-blind randomized placebo-controlled study. Biomed Rep.

[95] Karimian J, Hadi A, Pourmasoumi M, Najafgholizadeh A, Ghavami A. The efficacy of propolis on markers of glycemic control in adults with type 2 diabetes mellitus: a systematic review and meta-analysis. Phytother Res. 2019;33(6):1616–26. 10.1002/ptr.6356 30950136

[96] Pérez N, Moisan J, Sirois C, Poirier P, Grégoire J-P (2009). Initiation of insulin therapy in elderly patients taking oral antidiabetes drugs. Cmaj..

[97] Li Y, Chen M, Xuan H, Hu F. Effects of encapsulated propolis on blood glycemic control, lipid metabolism, and insulin resistance in type 2 diabetes mellitus rats. Evid Based Complement Alternat Med. 2012;2012:1–8. 10.1155/2012/981896 PMC3118452 21716678

[98] Zamami Y, Takatori S, Koyama T, Goda M, Iwatani Y, Doi S (2007). Effect of propolis on insulin resistance in fructose-drinking rats. Yakugaku Zasshi.

[99] Aoi W, Hosogi S, Niisato N, Yokoyama N, Hayata H, Miyazaki H (2013). Improvement of insulin resistance, blood pressure and interstitial pH in early developmental stage of insulin resistance in OLETF rats by intake of propolis extracts. Biochem Biophys Res Commun.

[100] Kajimoto Y, Kaneto H. Role of oxidative stress in pancreatic β-cell dysfunction. Mitochondrial Pathogenesis. Berlin, Heidelberg: Springer; 2004. p. 168–76.

[101] Matsui T, Ebuchi S, Fujise T, Abesundara KJ, Doi S, Yamada H (2004). Strong antihyperglycemic effects of water-soluble fraction of Brazilian propolis and its bioactive constituent, 3,4,5-tri-O-caffeoylquinic acid. Biol Pharm Bull.

[102] Sameni HR, Ramhormozi P, Bandegi AR, Taherian AA, Mirmohammadkhani M, Safari M (2016). Effects of ethanol extract of propolis on histopathological changes and anti-oxidant defense of kidney in a rat model for type 1 diabetes mellitus. J Diabetes Investig.

[103] Murata K, Yatsunami K, Fukuda E, Onodera S, Mizukami O, Hoshino G (2004). Antihyperglycemic effects of propolis mixed with mulberry leaf extract on patients with type 2 diabetes. Altern Ther Health Med.

